# Basigin-2 upregulated by receptor activator of NF-κB ligand enhances lung cancer-induced osteolytic lesions

**DOI:** 10.1186/s12935-016-0302-9

**Published:** 2016-04-02

**Authors:** Cheng-Gong Liao, Li Yao, Wei Xie, Lili Liu, Sheng-Da Wu, Ning Lu, Jian-Guo Huang, Ling-Min Kong, He-Long Zhang

**Affiliations:** Department of Oncology, Tangdu Hospital, Cancer Institute, Fourth Military Medical University, Xi’an, 710038 People’s Republic of China; Department of Oncology, Urumqi General Hospital of Lanzhou Military Command of PLA, Urumqi, 830000 People’s Republic of China; Department of Pathology, Tangdu Hospital, Fourth Military Medical University, Xi’an, 710038 People’s Republic of China; Cadet Brigade, Fourth Military Medical University, Xi’an, 710032 People’s Republic of China; Cell Engineering Research Center and Department of Cell Biology, National Translational Science Center for Molecular Medicine, Fourth Military Medical University, Xi’an, 710032 People’s Republic of China

**Keywords:** Basigin-2, Lung cancer, Bone metastasis, Osteolytic lesion, RANKL

## Abstract

**Background:**

Lung cancer bone metastasis causes poor prognosis. Basigin-2, a novel cancer-associated biomarker, is upregulated in lung cancer and has been linked with tumor progression. But little is known about the role of basigin-2 in lung cancer bone metastasis and osteolytic lesion.

**Methods:**

Basigin-2 expression was evaluated in biopsy tissue specimens of 20 lung cancer patients with bone metastases via immunohistochemistry. Invasion assay and MTT proliferation assay were performed to test the invasion and proliferation of lung cancer cell after modulated basigin-2 expression. The osteoclastic activity of basigin-2 was detected in tibia cancer model by injected of lung cancer cells. The regulation role of receptor activator of NF-κB ligand (RANKL) on basigin-2 and its downstream molecules were measured by real-time quantitative RT-PCR, gelatin zymography and western blot analysis.

**Results:**

We found that basigin-2 was highly expressed in lung cancer bone metastases. Then, we demonstrated that basigin-2 could promote lung cancer cells invasion, metastasis and proliferation through upregulating metalloproteinases-2 (MMP-2), MMP-9 and vascular endothelial growth factor (VEGF) expression. The lung cancer cells overexpressing basigin-2 strongly induced the osteolytic lesions in immunodeficient mice, which were reduced by treatment with basigin-2 blocking antibody. Furthermore, we explored the enhanced basigin-2 molecular mechanism in lung cancer bone metastasis. Our results indicated the RANKL, pivotal for the control of bone resorption, could increase basigin-2 and its downstream molecules MMP-2, MMP-9 and VEGF expression in vitro.

**Conclusions:**

Basigin-2 upregulated by RANKL induces MMPs and VEGF, which may increase lung cancer cell metastasis ability and support osteoclastic activity. Thus, our data suggest important roles for basigin-2 in lung cancer-induced osteolytic lesion and implicate this protein potential application as a target for lung cancer bone metastasis therapy.

**Electronic supplementary material:**

The online version of this article (doi:10.1186/s12935-016-0302-9) contains supplementary material, which is available to authorized users.

## Background

Lung cancer is the most frequent cause of cancer-related death in the world, with about 15 % 5-year survival rate of patients with non-small cell lung cancer (NSCLC) [1] and 6 % of small cell lung cancer (SCLC) [2]. In the United States, it is expected that 224,210 people will receive diagnoses of lung and bronchial cancer and that 159,260 will die of the disease in 2014 [3, 4]. One of the characteristics of lung cancer is its high potential for invasion and metastasis. Bone metastasis is one of the most frequent sites of secondary lesions in lung cancer [5]. When the bone metastases established, the chance of survival as well as the quality of life dramatically drop, with intractable pain, nerve compression syndromes, increased risk of fractures, and hypercalcaemia [6]. Lung cancer bone metastasis is usually osteolytic in nature and relies on the ability of tumor cells to activate osteoclast bone resorption, which is a pivotal event in the disruption of bone and in the creation of physical space into which the tumor intrudes [7–9]. Therefore, it is significant to study the molecular mechanism of bone metastasis in lung cancer.

Basigin-2, also known as CD147, is a transmembrane glycoprotein present on the cancer cell surface belonging to the immunoglobulin superfamily, which was found to induce expression of matrix metalloproteinases (MMPs), particular MMP-2 and MMP-9 in fibroblasts or tumor cells. Several laboratories have subsequently identified the basigin-2 molecule in different origins of human cells and tissues, designating it the names of extracellular matrix metalloproteinase inducer (EMMPRIN) [10], HAb18G [11], and M6 antigen [12]. Several data have clearly shown a key role for basigin-2 in tumor progression and metastasis [13, 14]. Basigin-2 is also able to induce MMPs production in tumor cells in an autocrine fashion [15] and to enhance in vivo tumor angiogenesis by upregulating VEGF [16].

Since, our previous work have showed the up-regulation of basigin-2 in lung cancer [17]. The aim of this study was to test the potential involvement of basigin-2 in the ability of lung cancer cells to induce osteolytic lesions in bone. In this study, we found that basigin-2 was highly expressed in the lung cancer bone metastases. We also demonstrated that basigin-2 could upregulate MMP-2, MMP-9 and VEGF expression. In addition, overexpression of basigin-2 promoted the invasion, metastasis and proliferation in lung cancer cell. The immunodeficient mice, which were injected with lung cancer cell overexpressing basigin-2, had a greater ability to induce the development of radiographically detectable osteolytic lesions. But this effect was reduced by treatment with basigin-2 blocking antibody. Furthermore, we explored the enhanced basigin-2 molecular mechanism that involved the activation of the receptor activator of NF-κB ligand (RANKL) pathway, pivotal for the control of bone resorption. Thus, our data suggest important roles for basigin-2 in supporting osteoclastic activity and increased incidence of lung cancer bone metastases.

## Methods

### Tissue specimens and immunohistochemical analysis

Twenty biopsy tissue specimens of lung cancer bone metastases were collected from Tangdu Hospital of Fourth Military Medical University (FMMU, Xi’an, China) and were histologically confirmed by staining with hematoxylin and eosin (H & E). All individuals provided written informed consent, and the study was approved by the Hospital Ethics Committee.

Immunohistochemistry was performed using the Histostain-SP kit (Invitrogen, Carlsbad, CA, USA) according to the manufacturer’s manual. Anti-basigin-2 antibody was previously prepared using hybridoma technique in our laboratory, which was a murine monoclonal antibody with a clone number as HAb18, targeting Ig-C2 domain of human basigin-2 [18–20]. Immunohistochemistry was performed using a basigin-2 diagnostic kit (Jiangsu Pacific Meinuoke Biopharmaceutical Company) according to the manufacturer’s manual [21]. Immunopositivity was independently evaluated by two pathologists. In case of discrepancy, the two pathologists will re-review the slides together to reach a consensus. Detailed evaluation of IHC staining score was according to our previous work [13, 17, 22].

### Cell lines and culture conditions

The human lung cancer cell line A549 (lung adenocarcinoma cells) was obtained from the Type Culture Collection of the Chinese Academy of Sciences (Shanghai, China). The A549 cell was routinely cultured in RPMI-1640 medium (Hyclone Laboratories, Logan, UT, USA) supplemented with 10 % fetal calf serum (Gibco, Rockville, MD, USA) at 37 °C in a humidified atmosphere of 5 % CO2. The cell line authentication was assessed using short tandem repeat (STR) DNA profiling method every year in our laboratory and the latest verification was done in March 2014.

To obtain the conditioned medium from osteoblasts, primary cultures of calvarial osteoblasts were prepared by a modification of the sequential collagenase/trypsin digestion method [23, 24]. In brief, calvaria were removed from 7 to 9-day-old CD1 mice, cleaned free from soft tissue, and digested with 1 mg/mL *Clostridium histolyticum* type IV collagenase and 0.025 % trypsin for 20 min at 37 °C in HBSS with gentle agitation. The procedure was repeated three times and cells from the second and third digestions were plated in petri dishes and grown to confluence in DME supplemented with antibiotics and 10 % FCS. At confluence, cells were trypsinized by the standard procedure and plated in wells for experiments. The cells obtained with this method were positive for alkaline phosphatase (ALP) activity and expression of the osteoblast markers [24]. Then, cells were grown in DMEM plus 10 % FBS until 80 % confluence. The media were then replaced with serum-free media, and after 48 h, supernatants were collected, centrifuged, and stored at −80 °C until use. For the experiments of RANKL inhibition, primary osteoblasts were treated with 100 ng/mL osteoprotegerin (OPG), then after 48 h, supernatants were collected, centrifuged, and stored at −80 °C until use.

### Vector construction, stable transfection and siRNA

The coding regions of basigin-2 was inserted into pcDNA3.1 (Invitrogen, Carlsbad, CA, USA) [20]. Stable transfectant was screened with G418 (Calbiochem, San Diego, CA) after transfection. siRNAs targeting basigin-2 and scrambled negative control siRNA (SNC) were purchased from Invitrogen [13]. Next, we constructed the shBasigin-2 vevtor containing small hairpin RNA (shRNA) targeting basigin-2 mRNA. The stable shRNA transfection A549 cells were screened with purine (Calbiochem) [25].

### Real-time quantitative RT-PCR

Real-time quantitative RT-PCR was performed as described previously [26]. Expression data were uniformly normalized to glyceraldehyde-3-phosphate dehydrogenase (GAPDH) as an internal control, and the relative expression levels were evaluated using the *ΔΔ*Ct method [20, 27]. Primers were used as described previously [13, 14, 20].

### Western blot analysis

Cells were lysed with RIPA buffer (Beyotime, NanTong, China). Equal amounts (10 μg) of total protein were loaded, and then subsequently immunoblotted with the primary antibodies, including anti-basigin-2, VEGF and tubulin monoclonal antibodies (Santa Cruz, CA, USA). Proteins were detected using the Amersham enhanced chemiluminescence system (Pierce, Rockford, IL, USA) according to the manufacturer’s instructions.

### Gelatin zymography

Gelatin zymography was performed using 10 % SDS-PAGE containing 1 mg/mL gelatin. Transfected A549 cells (1.0 × 10^5^ per well) were cultured in 96-well plates. After attachment, the cells were washed and incubated in serum-free medium for 12 h. Then, the supernatants were collected and prepared in non-reducing loading buffer. After electrophoresis, SDS was removed using 2.5 % Triton X-100 to renature gelatinases. Gels were then incubated at 37 °C for 16 h in developing buffer (50 mM Tris–HCl (pH 7.8), 200 mM NaCl, 5 mM CaCl_2_, and 0.02 % Brij-35) and then stained with Coomassie Blue R-250. MMPs activity was visualized as clear bands against the blue-stained gelatin background.

### In vitro invasion assay and migration assay

MilliCell (12 mm diameter with 8 µm pores) chambers (Millipore Corporation, Billerica, MA, USA) were pre-coated with Matrigel (BD, Bedford, MA, USA) on the upper side. A total of 1 × 10^5^ serum-starved A549 cells were added to the upper compartment in medium supplemented with 0.1 % serum, and the chambers were placed into 24-well plates with medium containing 10 % serum. After 24 h at 37 °C, invaded cells on the lower membrane surface were fixed and stained with 0.1 % crystal violet. Invasive activity was quantified by counting nine high-power fields (HPFs, 400×) per chamber. Mean values were obtained from at least three individual chambers for each experimental point per assay. The migration assay was the same as the invasion assay, except that no Matrigel was used and the cell permeating time was 12 h.

### Wound-healing assay 

1 × 10^6^ cells were seeded in six-well plates, cultured overnight, and transfected with basigin-2 siRNA, basigin-2/pcDNA3.1 or scrambled negative control (SNC), respectively. When the culture had reached nearly 90 % confluency, the cell layer was scratched with a sterile plastic tip and then washed with culture medium twice and cultured again for up to 24 h with serum-reduced medium containing 1 % FBS. At different time points, photographic images of the plates were acquired under a microscope and the data were summarized based on sextuple assays for each experiment.

### Cell proliferation assay

Cells were plated in sextuplicate in 96-well plates (2 × 10^3^ per well) in 100 μL complete medium and allowed to attach overnight. 3-(4,5-dimethyl-2-thiazolyl)-2,5-diphenyl-2H-tetrazolium bromide (MTT) (20 μL at 5 mg/mL; Sigma, St. Louis, MO, USA) was added at different time points (24 h interval) for the different groups and then incubated for 4 h. The supernatant was discarded, the precipitate was dissolved in 200 μL dimethyl sulfoxide (DMSO), and plates were read with a microplate reader at 570 nm [28].

### Tibia cancer model by injected of lung cancer cells

Female BALB/c nude mice at 4–6 weeks of age were provided by the Laboratory Animal Research Center of FMMU, and the animal study was reviewed and approved by the Animal Care and Use Committee of FMMU. Mice were anaesthetized with i.p. injection of pentobarbital. A syringe with a 26 1/2 G needle was subsequently inserted in the proximal end of the tibia, and tumor cells (5 × 10^4^/10 μL Matrigel) were injected into the intramedullary space [29]. Radiographs were taken every week after injection using Carestream MS FX Pro in vivo imaging system (Carestream Health, Cheektowaga, NY, United States) [14]. Osteolytic lesions were identified on radiographs as radio-opaque regions. For the antibody blocking treatment, 5 μg of basigin-2 antibody [18], or negative control IgG were injected twice weekly until the end of the experiment.

### Statistical analysis

All statistical analyses were performed using the SPSS statistical software package (version 16.0, Chicago, IL, United States). Each in vitro quantitative test was independently replicated, and all data are presented as mean ± SEM. All the statistical tests were two sided, and *P* < 0.05 was considered with statistical significance.

## Results

### High expression level of basigin-2 in lung cancer bone metastases

**Fig. 1:**
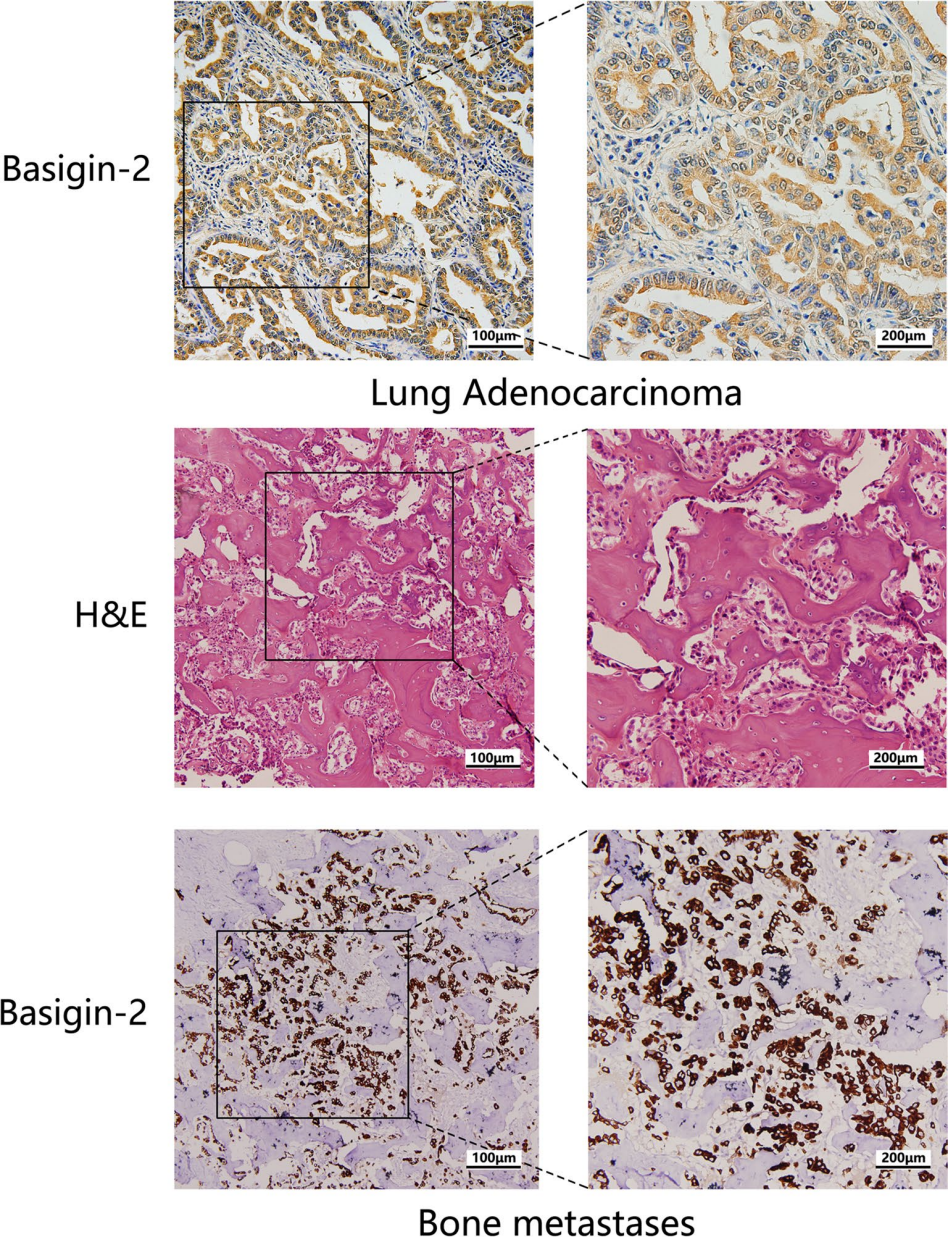
High expression of basigin-2 in lung cancer bone metastases. Representative immunohistochemistry analysis of basigin-2 expression in lung cancer tissues and the bone metastases. The *left panel*, magnification, ×200. The *right panel* is the amplification with magnification ×400. Top, the basigin-2 was high expressed in the lung adenocarcinoma. The *middle* and *bottom* were bone metastases of the lung cancer patient detected by H & E and IHC staining

In the first set of experiments, basigin-2 expression was evaluated in biopsy tissue specimens of 20 lung cancer patients with bone metastases via immunohistochemistry. As showed in Fig. 1, basigin-2 protein was predominantly localized in tumor epithelial cells, whereas little was detected in stroma. We also found that basigin-2 displayed positive membranous and cytoplasmic staining. Basigin-2 positive expression found in lung cancer bone metastases was 65 % (13/20). However, basigin-2 expression was not detected in the bone tissues of lung cancer patients without bone metastasis. So our results indicated that basigin-2 was highly expressed in lung cancer bone metastases, which may participate in the cancer distant metastasis.

### Characterization of lung cancer cells with modulated basigin-2 expression

**Fig. 2:**
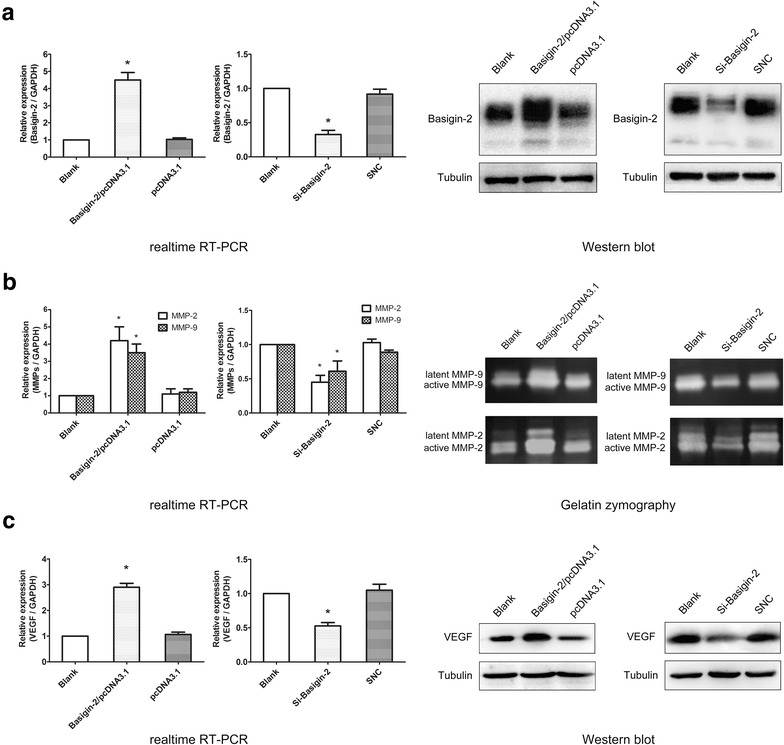
The regulation effect of basigin-2 on MMP-2, MMP-9 and VEGF expression in lung cancer cell. **a** The mRNA and protein expression of basigin-2 in A549 cells transfected with overexpression vector or siRNA detected by realtime RT-PCR and western blot, respectively. SNC means scrambled negative control of siRNA. After modulating basigin-2 expression, the downstream molecular MMP-2, MMP-9 (**b**) and VEGF (**c**) mRNA and protein expression level (proteinase activity for MMPs) were detected using realtime RT-PCR, gelatin zymography and western blot in A549 cells. *P < 0.05, compared to negative control (pcDNA3.1 or SNC), by Student’s *t* test

To explore the effect of modulated basigin-2 expression in lung cancer cell, we transfected the A549 cell with basigin-2 overexpression vector or siRNA, respectively. As showed in Fig. 2a, we exhibited that the ectopic expression or siRNA knockdown, respectively, increased or reduced basigin-2 mRNA and protein expression. Modulated basigin-2 expression were accompanied by a significant increase or decrease of MMP-2 and MMP-9 mRNA expression and proteinase activity (Fig. 2b). In addition, the expression of both VEGF mRNA and protein were significantly upregulated or downregulated in A549 cells (Fig. 2c).

**Fig. 3:**
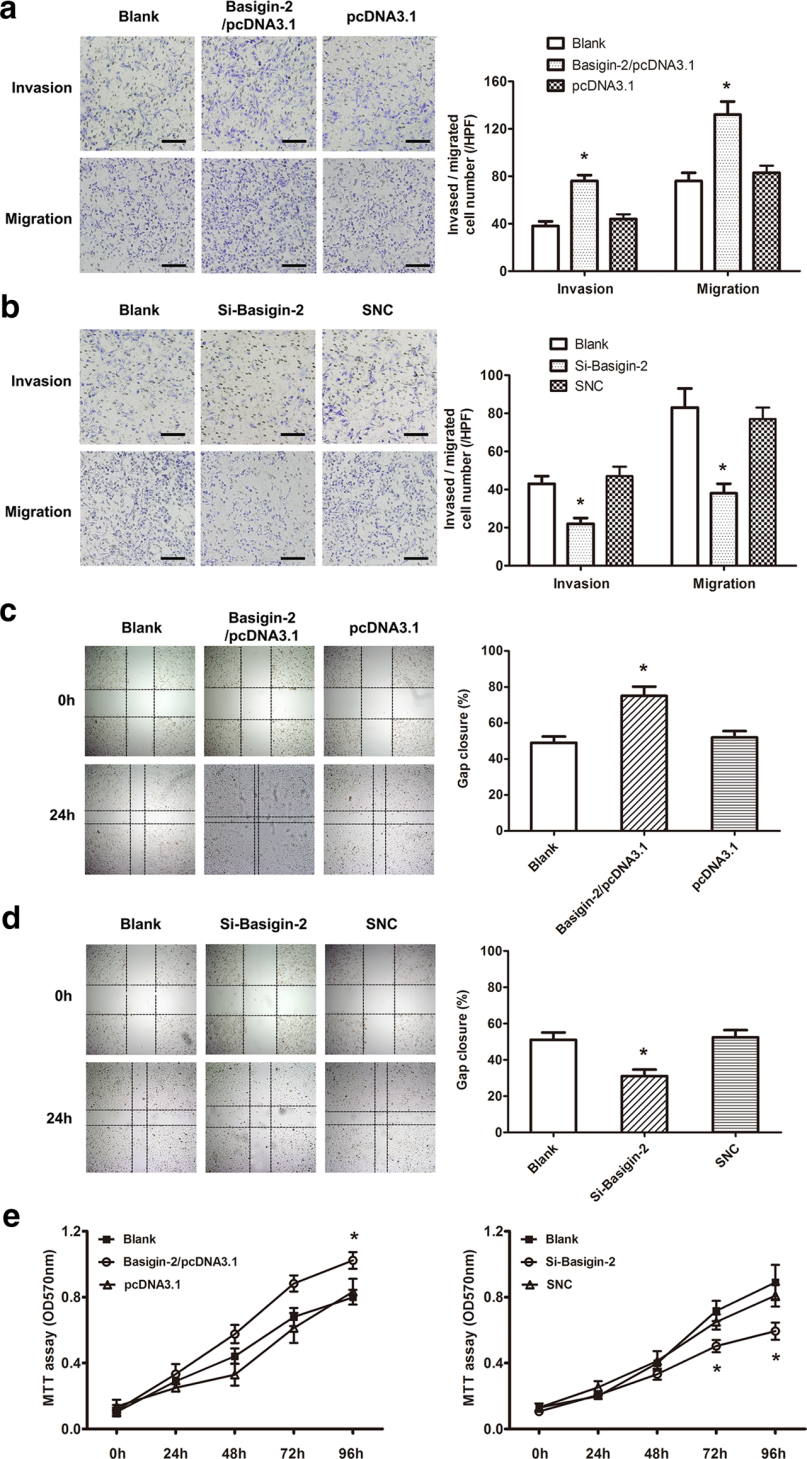
The furtherance role of basigin-2 on lung cancer cell invasion, metastasis and proliferation. **a** Effect of basigin-2 on the invasion and migration of A549 cells transfected with basigin-2 expression plasmid. **b** The invasion and migration of A549 cells transfected with basigin-2 siRNA were tested. SNC means scrambled negative control of siRNA. Morphologic comparison of cells penetrating the artificial basement membrane was shown.** c** and** d** Wound-healing assay of A549 cells transfected with basigin-2 expression plasmid or siRNA, respectively. *P < 0.05, compared to negative control (pcDNA3.1 or SNC), by one-way ANOVA followed by the Dunnett test. *Scale bars* 200 μm. **e** Cell proliferation of lung cancer cells transfected as above (**a**) and (**b**) was measured in the indicated time periods using MTT assay. *P < 0.05, compared to negative control (pcDNA3.1 or SNC), by 2-way repeated measures ANOVA followed by the Bonferroni test

Based on above results, we detected whether basigin-2 could change the capacities of lung cancer cells for migration, invasion and proliferation. As expected, transfection of basigin-2 expression plasmid into A549 cells resulted in increased migration rate and invasion rate compared with the control (Fig. 3a). In contrast, transfected with siRNA displayed the opposite results (Fig. 3b). Next, the wound-healing assay showed that A549 cells with overexpression of basigin-2 presented a quicker closing of scratch wound, compared with the controls (Fig. 3c). Transfection with siRNA showed the opposite result (Fig. 3d). The lung cancer cell proliferation was also promoted by modulated basigin-2 expression (Fig. 3e). Our results indicated that basigin-2 could regulate the downstream molecules MMP-2, MMP-9 and VEGF expression and contributed to a promotion in migration, invasion and proliferation of lung cancer cells.

### The promotion effect of basigin-2 on the osteolytic lesions

**Fig. 4:**
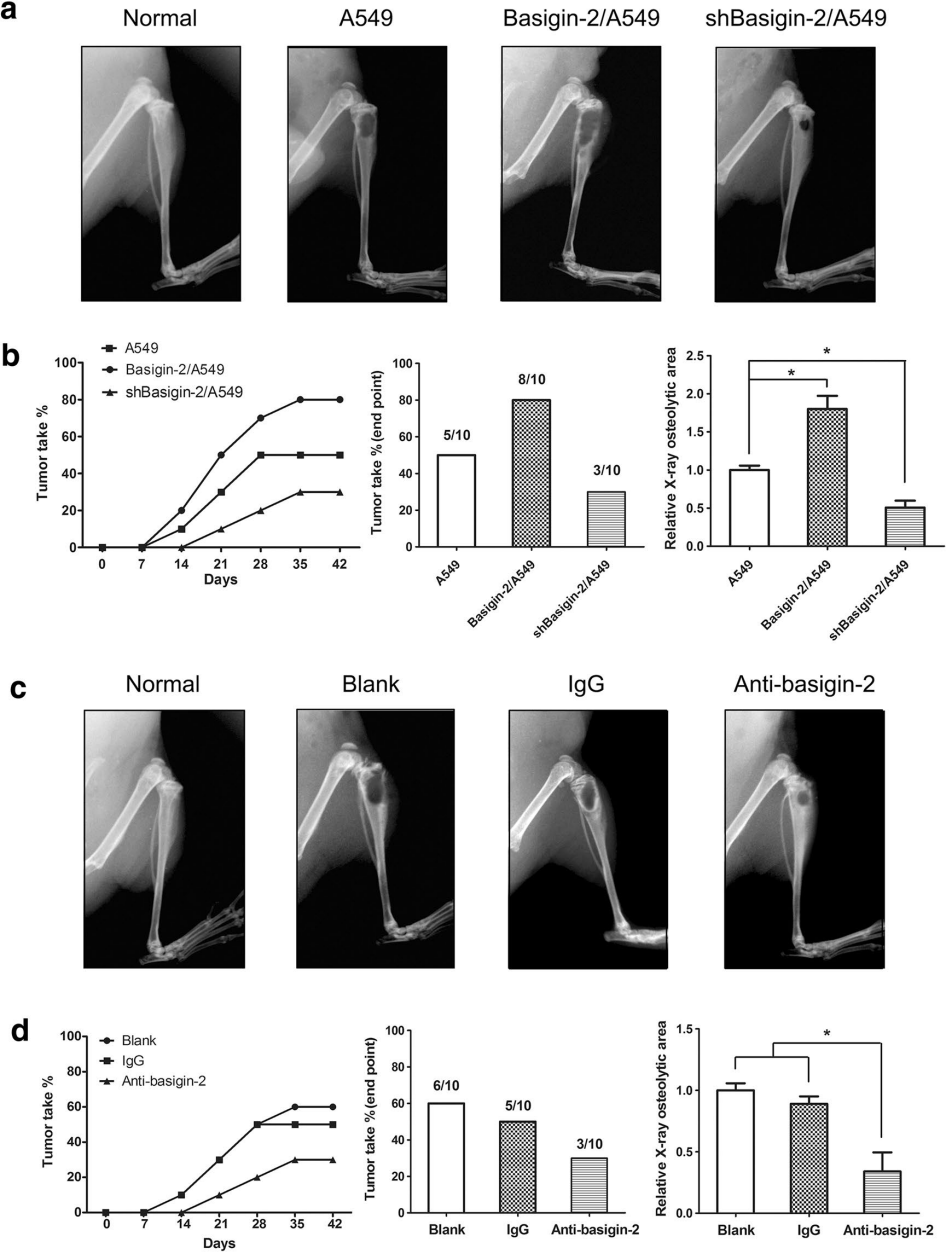
The promotion effect of basigin-2 on the osteolytic lesions. **a** The X-rays of the hind limb of mice injected with A549, basigin-2/A549 or shbasigin-2/A549 cells compared with normal mouse. **b** Evaluation of the incidence of tumor take (*left two panel*) and bone densitometric analysis of the radiographs assessing the osteolytic areas taken at the end point of the experiment (*right panel*). Inset numbers, number of tibia developing osteolytic lesions mice/number of tibia injected mice. *P < 0.05, by Student’s *t* test. **c** The X-rays of the tibia of mice injected with basigin-2/A549 cells treated using basigin-2 blocking antibody compared with IgG control. **d** Evaluation of the incidence of tumor take (*left two panel*) and bone densitometric analysis of the radiographs assessing the osteolytic areas taken at the end point of the experiment (*right panel*). Inset numbers, number of tibia developing osteolytic lesions mice/number of tibia injected mice. *P < 0.05, by Student’s *t* test

The lung cancer A549 cells used in our experiments were highly osteotropic, and when injected into the bone of immunodeficient mice, they grew causing osteolytic lesions that increased over time in a progressive fashion (Fig. 4a middle panel). Thus we compared the effects of A549 cells with overexpression of basigin-2, downexpression of basigin-2 and A549 empty vector cells after their inoculation into the tibia of nude mice. When injected with basigin-2 stable overexpression A549 cells, the incidence and extension area of osteolytic lesions were significantly higher in nude mice than the empty vector control (Fig. 4a, b). Downregulation of basigin-2 showed the opposite results (Fig. 4a right panel). So our results indicated the basigin-2 could significantly promote the lung cancer osteolytic lesions.

To validate the notion, mice intratibially inoculated with A549 cells were treated with a basigin-2 blocking antibody [30]. Our results exhibited that the ability of the blocking antibody to reduce the incidence and extension area of osteolytic lesions (Fig. 4c, d). There was no significant difference in mice body weight among the four groups of different treatment (Additional file 1: Figure S1). Our above results have indicated that basigin-2 could promote the lung cancer cells migration and invasion. Hence, these results suggest that basigin-2 play important roles in lung cancer bone metastasis induced osteolytic lesions.

### The regulation role of RANKL on basigin-2 expression

**Fig. 5:**
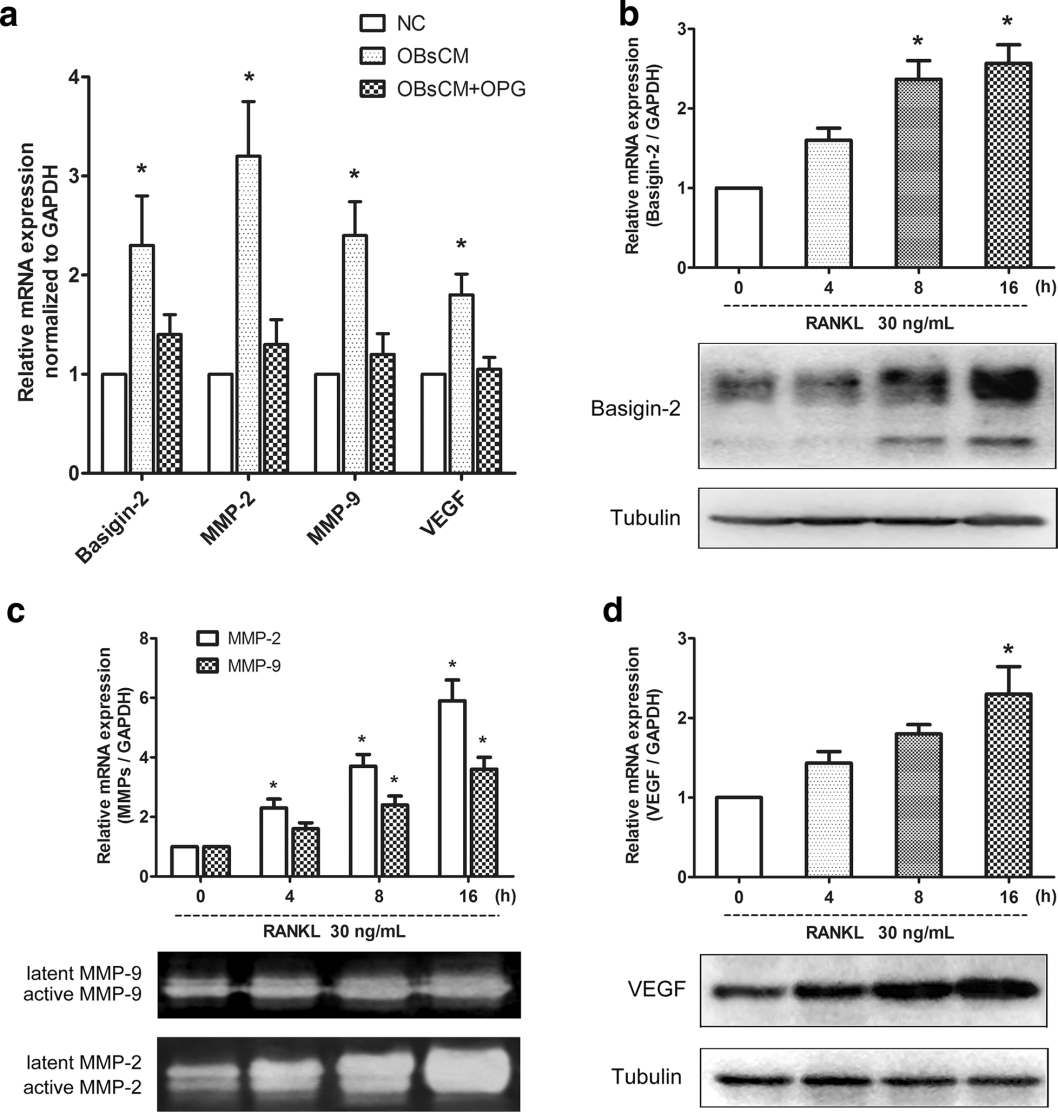
Effect of RANKL on the expression of basigin-2 and its downstream genes. **a** A549 cells were treated with DMEM (negative control, NC) or with conditioned medium from primary mouse osteoblast cultures treated with vehicle (OBsCM) or OPG (OBsCM + OPG) for 48 h. The mRNA expression of basigin-2, MMP-2, MMP-9 and VEGF were detected by realtime RT-PCR. *P < 0.05, compared to negative control (NC), by Student’s *t* test. **b**–**d** A549 cells were treated with 30 ng/mL RANKL for 4, 8, and 16 h. Then, the mRNA and protein expression level were measured for **b** basigin-2, **d** VEGF and proteinase activities for **c** MMP-2 and MMP-9. *P < 0.05, compared to 0 h, by Student’s t test

To unravel the regulatory mechanisms of basigin-2 promoting osteolytic lesion, the A549 cells were exposed to conditioned medium from osteoblasts (OBsCM). The medium contains the osteoblast markers alkaline phosphatase, runt-related transcription factor 2, parathyroid hormone/parathyroid hormone–related peptide receptor, and type I collagen and osteocalcin, which could produce paracrine effectors of lung cancer growth into the bone [14]. Notably, the expression levels of basigin-2, MMP-2, MMP-9 and VEGF were increased by the OBsCM than the control (Fig. 5a). Previous works have suggested that RANKL is a key inducer of osteolytic bone metastasis through its pro-osteoclastogenic activity. There was RANK expression in A549 cells [31]. So we detected the effect of RANKL on the basigin-2 expression regulation. Interestingly, the upregulated expression of basigin-2, MMP-2, MMP-9 and VEGF induced by OBsCM were abolished through treating with OPG, a decoy receptor that physiologically inhibited RANKL activity (Fig. 5a).

To further prove the role of RANKL in modulating basigin-2 and its downstream genes, the A549 cells were treated with recombinant RANKL. As showed, the basigin-2 mRNA and protein expression level were increased in a time-dependent pattern (Fig. 5b). The downstream genes, MMP-2, MMP-9 and VEGF were also increased by RANKL (Fig. 5c, d). A parallel increase of MMP-2, MMP-9 activity (Fig. 5c, inset) and VEGF protein expression (Fig. 5d, inset) was seen after treatment with RANKL. Hence, our results suggest that RANKL may through basigin-2 pathway enhance osteoclast activity.

## Discussion

In our study, we showed that basigin-2 was highly expressed in the lung cancer bone metastases and could regulate the downstream molecules MMP-2, MMP-9 and VEGF expression, which promoted migration, invasion and proliferation of lung cancer cells. When injected with basigin-2 stable overexpression A549 cells in the tibia of nude mice, the incidence and extension area of osteolytic lesions were significantly higher than those of empty vector control, which was reduced by treatment with basigin-2 blocking antibody. Furthermore, our results revealed that RANKL could increase basigin-2 and its downstream molecules MMP-2, MMP-9 and VEGF expression. So RANKL may through basigin-2 pathway enhance osteoclast activity.

Lung cancer has a high potential for metastasis and is normally diagnosed when the disease has already progressed locally or systemically because there are few symptoms in the early stages of the disease [32]. A recent study reported that 41 % of patients had distant disease at the time of the diagnosis and of these 38 % suffered bone metastasis during the first year following their cancer diagnosis [33]. Our previous work showed that basigin-2 was more strongly upregulated in lung cancer tissues than in the adjacent tissues [17]. Our results indicated that basigin-2 could regulate its downstream molecules MMP-2, MMP-9 and VEGF expression and promote migration, invasion and proliferation of lung cancer cells. One hallmark of metastasis and invasive growth is the transition of tumor cells from an epithelial to a mesenchymal morphology, known as the epithelial–mesenchymal transition (EMT). MMPs help cancer cells spread by breaking down the extracellular matrix (ECM) and other barriers MMP-2 and MMP-9 were previously reported to be involved in human tumorigenesis and cancer metastasis [34]. VEGF is a key regulatory factor in angiogenesis and vascular permeability in both physiological and pathological states [35]. Several studies have reported the association between the VEGF polymorphisms with lung cancer risk [36]. Our current data are in keeping with the known functions of basigin-2 in human malignancies and strengthen the key role played by this molecule in lung cancer progression.

Bone metastasis is a frequent complication of cancer, occurring in up to 70 percent of patients with advanced breast or prostate cancer and in approximately 15–30 percent of patients with carcinoma of the lung, colon, stomach, bladder, uterus, rectum, thyroid and kidney [37]. Our findings indicated that basigin-2 could significantly promote the lung cancer osteolytic lesions in vivo. Thus, basigin-2 plays important roles in lung cancer bone metastasis induced osteolytic lesions. The bone microenvironment plays a critical role in the formation of osteoclasts through the production of macrophage colony-stimulating factor and RANKL [38] by stromal cells or osteoblasts. RANKL binds to the RANK receptor on osteoclast precursors and induces the formation of osteoclasts by signaling through the nuclear factor-κB and Jun N-terminal kinase pathways [39]. In this study, RANKL could increase basigin-2 and regulate its downstream molecules. Herein, we have shown a link between tumor basigin-2 and the RANKL pathway. Increased RANKL expression by osteoblasts is a hallmark of bone metastases and is deemed to contribute to enhanced osteoclast activity at sites of osteolytic lesions [39]. Our work is consistent with that conditioned medium from primary osteoblasts enhanced basigin-2 expression in lung cancer cells. Therefore, our data suggest that the mechanism whereby basigin-2 induces osteolysis relies to a direct basigin-2 activity as well as to the induction of the release of downstream effectors MMPs and VEGF.

## Conclusion

To the best of our knowledge, this is the first study to examine the role of basigin-2 in lung cancer-induced osteolytic lesion and to reveal that a RANKL-basigin-2-MMPs/VEGF pathway plays an important role in this process. These findings will contribute to our understanding of the molecular mechanism of lung cancer-induced osteolytic lesion and might aid in an alternative target for bone metastasis therapy.

## Additional file

10.1186/s12935-016-0302-9 The body weight of mice among the four groups of different treatment. There was no significant difference in the four groups analyzed by 2-way repeated measures ANOVA followed by the Bonferroni test.
