# Supplementary material for: Basigin-2 upregulated by receptor activator of NF-κB ligand enhances lung cancer-induced osteolytic lesions
Source: Cancer Cell Int. 2016 Apr 2;16:28. doi: 10.1186/s12935-016-0302-9 (PMC4818914; doi:10.1186/s12935-016-0302-9)
Supplement: Supplementary file 1 — 10.1186/s12935-016-0302-9 The body weight of mice among the four groups of different treatment. There was no significant difference in the four groups analyzed by 2-way repeated measures ANOVA followed by the Bonferroni test. [file 12935_2016_302_MOESM1_ESM.docx]

**Supplementary Figure 1. The body weight of mice among the four groups of different treatment.** There was no significant difference in the four groups detected by by 2-way repeated measures ANOVA followed by the Bonferroni test.

**
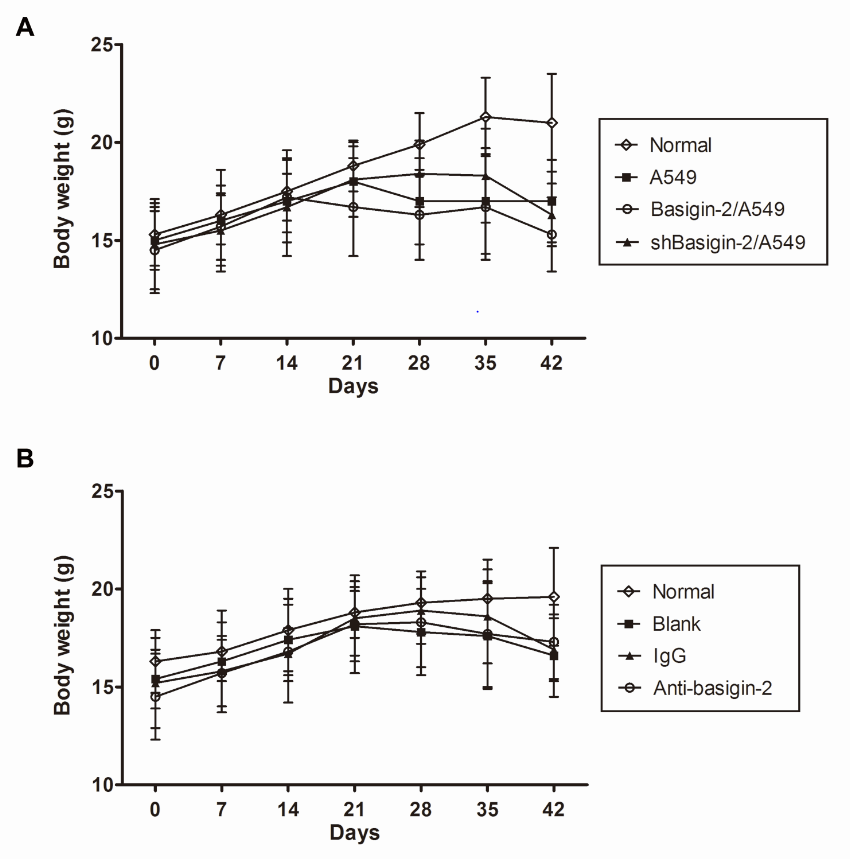
**
